# Isolation, characterization, and *in vitro *propagation of infantile hemangioma stem cells and an *in vivo *mouse model

**DOI:** 10.1186/1756-8722-4-54

**Published:** 2011-12-22

**Authors:** Dan Xu, Teresa M O, Archil Shartava, Taylor C Fowles, Jianchang Yang, Louis M Fink, David C Ward, Martin C Mihm, Milton Waner, Yupo Ma

**Affiliations:** 1 Department of Pathology, SUNY at Stony Brook, Stony Brook, NY 11794, USA; 2 Vascular Birthmark Institute of New York, New York, NY 10023, USA; 3 Division of Laboratory Medicine, Nevada Cancer Institute, 1 Breakthrough Way, Las Vegas, NV 89135, USA; 4 Cancer Research Center of Hawaii, University of Hawaii, Honolulu, HI 9681, USA; 5 Massachusetts General Hospital, Dermatopathology, Boston, MA 02114, USA; 6 Biopharmaceutical Research Center of Chinese Academy of Medical Sciences and Peking Union Medical College, Suzhou, China

**Keywords:** Infantile hemangioma, stem cells, tumor spheres, SALL4

## Abstract

**Background:**

Infantile hemangiomas (IH) are the most common benign tumors of infancy. The typical clinical course consists of rapid growth during the first year of life, followed by natural and gradual involution over a multi-year time span through unknown cellular mechanisms. Some tumors respond to medical treatment with corticosteroids or beta-blockers, however, when this therapy fails or is incomplete, surgical extirpation may be necessary. Noninvasive therapies to debulk or eliminate these tumors would be an important advance. The development of an *in vitro *cell culture system and an animal model would allow new insights into the biological processes involved in the development and pathogenesis of IH.

**Results:**

We observed that proliferative stage IH specimens contain significantly more SALL4+ and CD133+ cells than involuting tumors, suggesting a possible stem cell origin. A tumor sphere formation assay was adapted to culture IH cells *in vitro*. Cells in IH tumor spheres express GLUT1, indicative of an IH cell of origin, elevated levels of VEGF, and various stem/progenitor cell markers such as SALL4, KDR, Oct4, Nanog and CD133. These cells were able to self-renew and differentiate to endothelial lineages, both hallmarks of tumor stem cells. Treatment with Rapamycin, a potent mTOR/VEGF inhibitor, dramatically suppressed IH cell growth *in vitro*. Subcutaneous injection of cells from IH tumor spheres into immunodeficient NOD-SCID mice produced GLUT1 and CD31 positive tumors with the same cellular proliferation, differentiation and involution patterns as human hemangiomas.

**Conclusions:**

The ability to propagate large numbers of IH stem cells *in vitro *and the generation of an *in vivo *mouse model provides novel avenues for testing IH therapeutic agents in the future.

## Background

Infantile hemangiomas are benign tumors whose proliferative phase during the first year of life is characterized by a rapid outgrowth of vascular endothelial cells. An involuting phase then occurs lasting up to 10 years with replacement of vascular tissues by fibrofatty tissues[1-4]. Clinically, most hemangiomas present few serious health problems. In some cases, they may be extremely disfiguring, impede vision, cause airway obstruction or congestive heart failure [5-7]. Traditionally, medical therapy for IH involved the use of topical, intralesional or systemic corticosteroids. This has now largely been replaced by beta-blockers. When medical therapy fails or is incomplete, surgical resection is necessary [8,9].

Depending on the patient's age and degree of surgical resection, these vascular tumors may recur at the same location[10]. This suggests either incomplete surgical resection or the presence of a population of tumor stem cells that is responsible for recurrence[11]. The isolation of IH stem cells using anti-CD133 antibodies and immunomagnetic techniques was recently reported [12]. Transplantation of these cells into nude mice produced tumors that were composed of endothelial cells and blood vessels. However, while the formation of blood vessels was followed by involution and fibrofatty tissue production, no obvious proliferative phase was observed.

In the study reported herein, the isolation of IH stem cells was accomplished using growth in selective culture media. These cells form tumor spheres that express CD133 [13,14] and other stem/progenitor cell markers and possess self-renewal capabilities. The tumor sphere cells can be differentiated to GLUT1-expressing cells (indicative of an IH cell origin) [15-17] by exposure to VEGF.

By multiplex Luminex analysis, we demonstrated that a specific growth factor, VEGF, is secreted from the IH tumor spheres and that an mTOR/VEGF inhibitor, Rapamycin, dramatically inhibits IH tumor stem cell growth. Furthermore, when cells from tumor spheres are injected into nude mice, they recapitulate human IH tumors, exhibiting characteristic proliferative and involuted phases.

## Methods

### Patient Samples

Infantile hemangioma tissues were obtained with approval of Yale University Institutional Review Board and all participants gave informed written consent.

### Immunohistochemical staining

Staining was performed according to standard techniques as previously reported [18].

### IH Tumor Sphere Culture

Post-operative IH tissue samples were washed using PBS and transferred into a sterile Petri dish. The tissue was minced into a fine paste (1 × 1 mm^2^) and washed with PBS again. IH tissues were then digested with 2 mg/ml collagenase (Invitrogen, CA) for at least 2 hours shaking at 37°C. The cells were dispersed by repeat passage through a pipette tip and filtration through a 100 μM nylon cell strainer. Cells were plated on low attachment Petri dish at a density of 2 × 10^5 ^cells/ml using a stem cell culture (SCC) media (all ingredients from Invitrogen, CA) consisting of Knockout DMEM, 15% Knockout Serum Substitute, 1x non-essential amino acids (NEAA), and 20 ng/ml of both basic fibroblast growth factor (bFGF) and human endothelial growth factor (hEGF). Tumor spheres were passaged approximately every seven days (roughly 200 cells/sphere); spheres were washed once with PBS and then single cell suspension made by incubation with 2 mg/ml collagenase IV at 37°C for 10 minutes.

### Differentiation of IH Tumor Spheres

Tumor spheres were transferred to gelatin coated dishes to attach and were differentiated using IH differentiation medium containing Knockout DMEM, 15% Knockout Serum Substitute, 1x non-essential amino acids (NEAA), 20 ng/ml each of basic fibroblast growth factor (bFGF), 20 ng/ml human endothelial growth factor (hEGF), and 50 ng/ml vascular endothelial growth factor (VEGF) (R&D Minneapolis MN).

### Immunofluorescent staining

Cells were fixed with 4% freshly prepared paraformaldehyde for 15 minutes. Following three washes, the cells were blocked with 5% bovine serum albumin (BSA) in 1x phosphate buffered saline-Triton X-100 (TPBS) for 1 h at room temperature. Primary antibodies [mouse anti-GLUT1 (1:80 Abcam, Cambridge, MA), rabbit anti- GLUT1 (1:80, Abcam, Cambridge, MA), rabbit anti-mouse FDR (1:50, Alpha Diagnostic International, San Antonio, TX), rat-anti-mouse CD31 (1:25 BD Biosciences, San Jose, CA), rabbit anti-CD133 (1:25 Biocare Medical, Concord, CA) or rabbit anti-SALL4 (1:400; developed by this laboratory)] in TPBS/1% BSA were added to the tissue sections and the slides were incubated at 4°C overnight. Following a wash with TPBS, an appropriate secondary antibody [goat anti-rabbit-FITC (SouthernBiotech, Birmingham, AL), goat anti-rabbit-RPE (SouthernBiotech, Birmingham, AL), donkey anti-mouse-Rhodamine (Chemicon, Temecula, CA), or donkey anti-mouse-FITC (Sigma, Louis, MO)] was added and the slides were incubated at room temperature for 2 h in the dark. After another three washes with TPBS, the cells were counterstained with 50 ng/ml 4', 6-diamidino-2-phenylindole (DAPI) for 10 minutes.

### Measurement of growth factor secretion from tumor spheres

Tumor spheres were cultured in stem cell culture media for 15 days. The conditioned media, which had been exposed to the tumor cells for 5, 10 and 15 days, was collected and analyzed for the levels of the growth factors (VEGF, EGF, FGF basic and G-CSF) using the human growth factor (GF)4-Plex kit (Invitrogen, Carlsbad, CA) and following manufacture's recommended protocol.

### Rapamycin, a potent mTOR/VEGF inhibitor, suppresses proliferation of IH tumor stem cells *in vitro*

Tumor spheres were seeded onto Petri dishes and exposed to 1 nM, 2 nM, and 4 nM Rapamycin in stem cell culture media (see Methods, IH Tumor Sphere Culture) for 10 days. Approximately 30 tumor spheres were collected at each time point (0, 3, 6 and 10 days), dissociated by collagenase IV to single cells and counted for cell numbers. Each sample was assayed in triplicate.

### Mouse model

IH tumor spheres cultured for one week were dissociated by collagenase to single cells and resuspended in Matrigel at a concentration of 5 × 10^6 ^cells per ml. 200 μl (10^6 ^cells) was injected subcutaneously in both sides of flanks of immunodeficient NOD/SCID mice. Matrigel with PBS or 0.9% NaCl (without tumor cells) was injected into two additional subcutaneous sites as a control. After 10, 20 and 30 days, the mice were sacrificed, the tumor tissues excised, and fixed in formalin. Some tissue sections were stained with standard hematoxylin and eosin (H & E). Other IH tissue sections were analyzed using anti-Glut1 antibody and anti-CD31, a human endothelial cell marker, for their expression levels.

## Results

### Isolation and in vitro culture of hemangioma stem cells

Until now, there has been no adequate in vivo model to evaluate novel therapeutic modalities for IH. The ability to culture IH specimens would be an extremely valuable translational research tool. Recently, Khan and colleagues described an hemangioma stem cell population that expressed CD133 and, when transplanted into nude mice, produced tumors that shared many characteristics of hemangiomas but lacked an obvious proliferative stage[12].

**Figure 1:**
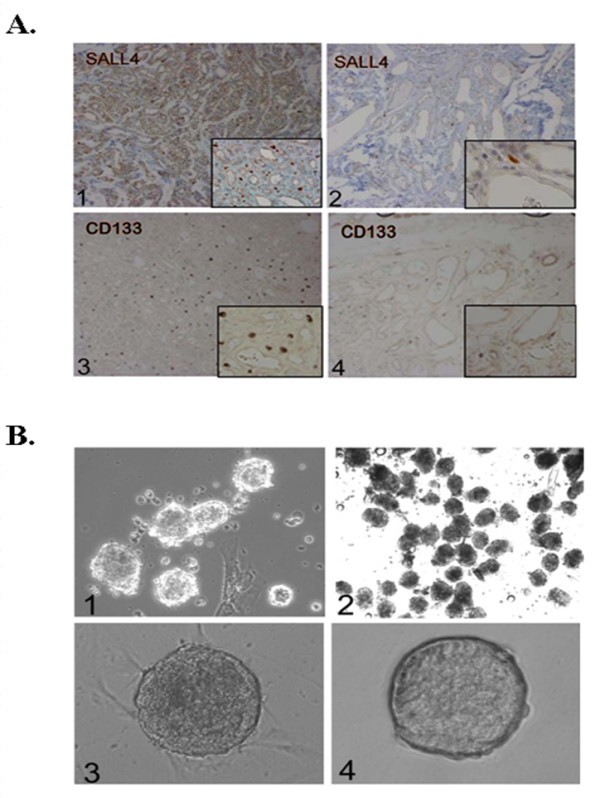
**Expression of SALL4 and CD133 in IH tissues**. (A) Immunohistochemistry staining of proliferative (1,3) and involuting (2,4) IH tissue sections with anti-SALL4 and CD133 antibodies. Magnification: 100x, inset 200x. (B) 1, Phase contrast image of IHs tumor spheres forming on Petri dishes with surrounding mature endothelial cells and/or fibroblasts. This was observed after 3 days of culture in serum-free media. Magnification: 100 ×. 2, the tumor spheres continue to grow under serum-free conditions while mature cells such as endothelial cells and fibroblasts do not. Magnification: 100x. 3, phase contrast image of high density tumor spheres in culture. Magnification: 100 ×. 4, comparison of IH tumor sphere (left) and induced pluripotent stem cell clone (right). Magnification: 100 ×.

Our group and others [19,20] have previously shown that mouse SALL4 plays an essential role in maintaining the self-renewal and pluripotent properties of embryonic stem (ES) cells and in governing the fate of the inner cell mass through transcriptional modulation of Oct4 (also known as *Pou5f1*) and Nanog [19-24]. We have also observed expression of SALL4 in hematopoietic stem cells suggesting a role in stem cells of various organ systems [25,26]. Very recently, we demonstrate that SALL4 acts as a robust stimulator for both human and mouse hematopoietic stem/progenitor cell *ex vivo *proliferation [27-29]. We have hypothesized that IHs are a stem cell disease and sought to determine if IHs express either SALL4 and/or CD133. By flow cytometry assay, it was shown that the proliferative IHs expressed both markers either alone or together (data not shown). Indeed, using immunohistochemistry, we observed significantly more cells expressing SALL4 and CD133 in proliferative IHs than in involuting phase IHs (Figure 1). This supports the hypothesis that IHs may involute because of depletion of stem cells or endothelial progenitor cell populations.

We next sought to establish an *in vitro *culture system to study the proliferation of IHs. Primary surgical specimens were dissociated and plated on Petri dishes in serum-free media. Mature endothelial cells and fibroblasts were unable to survive in these conditions. However, structures resembling the tumor spheres of other tumor types were able to grow as shown in Figure 1, 1. Tumor spheres derived from IH samples could be cultured for prolonged periods of time (currently up to passage 30). In addition, IH tumor spheres could also be expanded into high density cultures (Figure 1) - an advantageous feature for therapeutic uses. The IH tumor spheres also resembled induced pluripotent (iPS) stem cells that have been generated from fibroblasts in our laboratory (Figure 1, 1), and also express SALL4. This suggests that the tumor spheres may contain stem cells.

**Figure 2:**
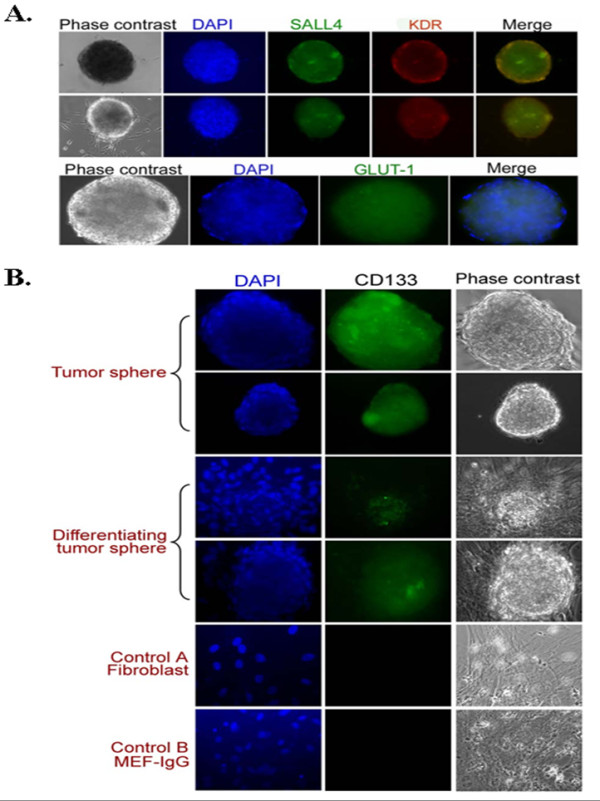
**Immunostaining of IH tumor spheres**. (A) Immunostaining of IH tumor spheres with antibodies specific for SALL4, FDR (top) and GLUT1 (bottom). Tumor spheres expressed these three markers. Mature cells surrounding tumor spheres were negative for SALL4 and FDR. Magnification: 100 ×. (B) Immunostaining of IH tumor sphere and differentiating tumor sphere with an anti-CD133 antibody. The anti-CD133 reacted specifically with tumor spheres but not differentiated cells. Magnification: 100 ×.

**Figure 4:**
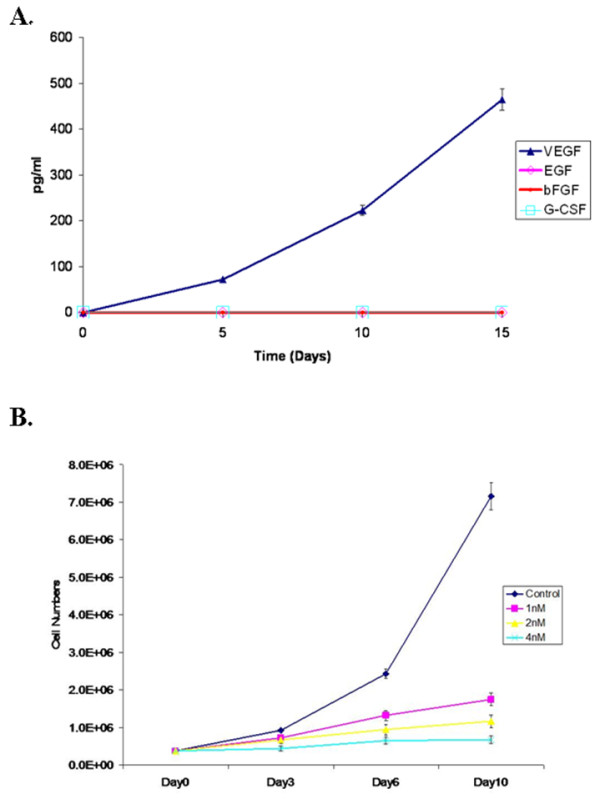
**IH tumor sphere specifically secrete VEGF and Rapamycin, an mTOR/VEGF inhibitor inhibits proliferation of IH tumor stem cells**. A, Concentration of VEGF secreted from tumor spheres at different days of cell culture. Every 5 days media was collected and the concentration of VEGF was evaluated by multiplex Luminex assay. Three independent measurements were performed. The result indicates that IH tumor spheres continuously secreted VEGF and that the secreted growth factor may be involved in the proliferation of the tumor spheres. B, Inhibition of growth of tumor spheres by Rapamycin. Three different concentration of Rapamycin, 1 nM, 2 nM and 4 nM was used. At days 0, 3, 6 and 10, a sample of tumor spheres was collected, dissociated with collagenase IV and number of cells were counted.

### Characterization of IH tumor spheres

Next, we wanted to ensure that spheres formed in our assay were enriched for stem/progenitor cell markers and that IHs were the cell of origin. As shown in Figure 2 and 2, these spheres were positive when immunostained with antibodies against human stem cell markers, SALL4 and CD133, and FDR (an early marker for endothelial cell precursors) as well as an IH signature marker, GLUT1. These data suggest that IH tumor spheres are enriched for stem cells or progenitor cells and that they express markers for early endothelial differentiation and are derived from IH tissue.

**Figure 3:**
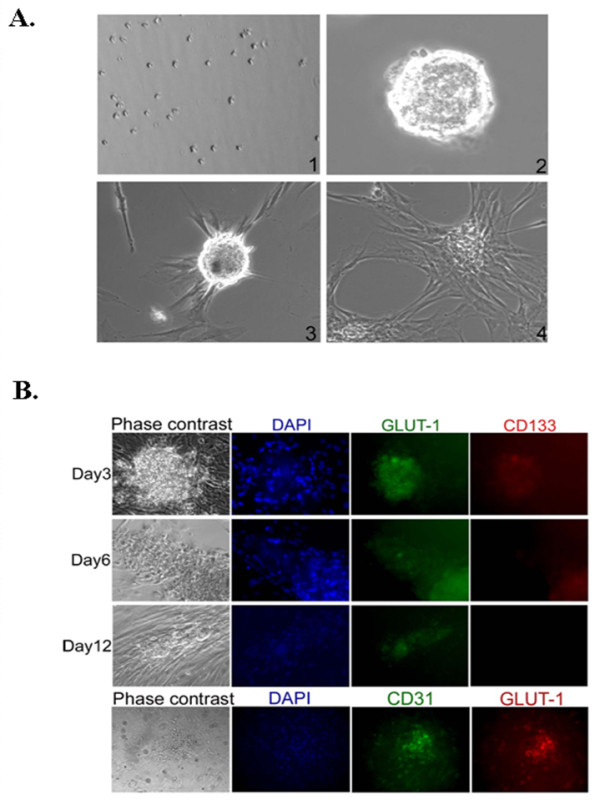
**Characterization of IH tumor spheres**. (A) Determination of the self renewal and differentiation capabilities for IH tumor spheres. 1) Dissociation of secondary tumor spheres to single cell colonies. 2) Tertiary tumor sphere formed from a single cell of a dissociated secondary tumor sphere. 3) Phase contrast image of differentiating tumor sphere in the presence of VEGF and its spider like morphology exhibited after 24 hours. 4) Tumor spheres differentiating into endothelial like structures after 5 to 6 days of culture in the presence of VEGF. Magnification: 100x. (B) Immunostaining of differentiating tumor spheres over time with anti-GLUT1 and anti-CD133 antibodies (top panel). The bottom panel represents fully differentiated tumor spheres. Magnification: 100 ×.

Since IH tumor spheres express markers for immature cell lineages and they are the IH cell of origin, we sought to examine if tumor spheres bear the stem cell properties of self renewal and differentiation. Following the preparation of single cell suspensions, replating of single cells from tumor spheres gave rise to secondary tumor spheres within 3 to 4 days. The process of dissociation to single cells was repeated and the formation of tertiary tumor spheres was observed (Figure 3 and 3) and tumor spheres could be passaged for prolonged periods of time (currently up to passage 30). This suggests that tumor spheres are able to self renew in the absence of differentiation signals. When tumor spheres were cultured in media favoring endothelial differentiation using VEGF, they would adhere and differentiate toward an endothelium- like morphology (Figure 3 and 3). The differentiating tumor spheres continually expressed GLUT1 (Figure 3). Expression of an endothelial marker, CD31, increased from day 3 to day 12 and expression of CD133 decreased over the same time period (Figure 3). This data illustrates that tumor spheres derived from IH tissues have stem cell properties and supports the hypothesis that IH is a stem cell disease.

### Tumor spheres specifically secrete VEGF (vascular endothelial growth factor)

Studies have indicated that tumor stem cells often contribute to or are associated with a microenvironmental niche. Understanding this process may allow development of a tumor stem cell therapy. We measured the concentration of growth factors, VEGF, EGF, FGF basic and G-CSF, in tumor sphere culture media at different time points (Figure 4). The level of the growth factors in the media was set as a background (blank). These values were automatically deducted by the software from the concentrations of the growth factors which were measured in the samples of interest. The concentrations were derived from the measured level of fluorescence in the samples by comparison with the fluorescence of the standard curve of known concentrations. The values for EGF, bFGF and G-CSF were below the range of the standard curve and considered as insignificant. Thus they are not presented on the graph. Media was collected on day 0, 5, 10 and 15 and analyzed for the level of growth factors by using the Human Growth Factor (GF) 4-Plex Kit (Invitrogen, Carlsbad, CA). The concentration of VEGF was undetectable at day 0 but became detectable at day 5 and was significantly increased during the 15-day measurement period (Figure 4). However, none of the other growth factors including EGF were detectable during the 15 days.

### Treatment with Rapamycin, a potent mTOR/VEGF inhibitor, dramatically inhibits proliferation of IH tumor stem cells *in vitro*

As shown above, IH tumor stem cells were able to specifically secrete VEGF and this study indicates that VEGF may play a significant role in tumor stem cell growth. Should this hypothesis be correct, inhibition of VEGF functions should inhibit IH stem cell growth [30-32]. In fact, treatment of IH tumor stem cells with Rapamycin leads to inhibition of the growth of IH stem cells in our tumor sphere culture system. As shown in Figure 4, three different concentrations of Rapamycin, 1 nM, 2 nM and 4 nM, all in a biologically significant range, inhibited IH stem cell growth in a dose dependent manner. At days 0, 3, 6, 10, tumor sphere samples were collected, dissociated with collagenase IV, and the number of cells counted. Treatment with Rapamycin dramatically inhibited IH tumor stem cell growth.

### *In vivo *mouse model

**Figure 5:**
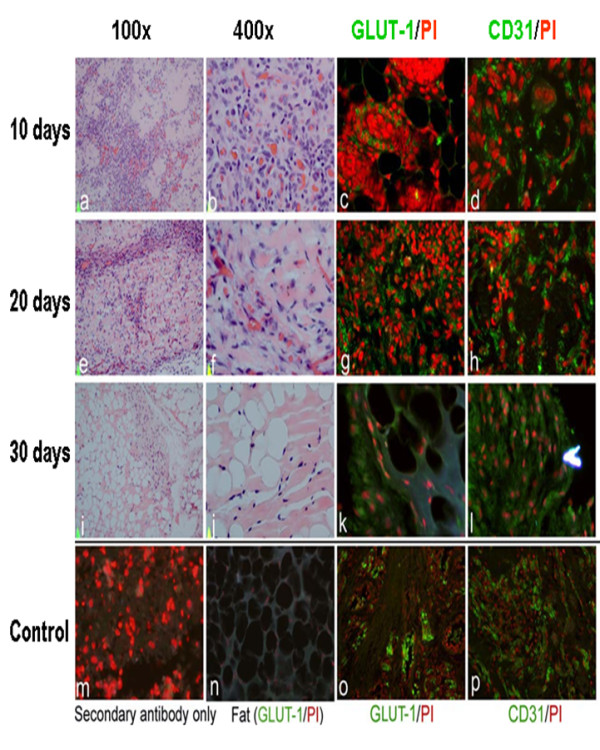
***In vivo *hemangioma mouse model**. Tissue sections of tumors formed after injection of cells from IH tumor spheres into mice were examined after 10, 20 and 30 days. (a, b, e, f, i, j) Hematoxylin and Eosin Staining (c, d, g, h, k, i) Immunofluorescent staining with anti-CD31 and anti-GLUT1 antibodies. Secondary antibody only (m) and mouse fat tissue staining with GLUT-1 (n) were used as negative control. Human hemangioma staining with GLUT-1(o) and CD31 (p) were used as positive controls.

In order to generate an *in vivo *hemangioma mouse model, and to determine whether IH stem cells have the ability to generate tumors in immunodeficient mice, dissociated IH tumor sphere cells were resuspended in Matrigel and injected subcutaneously into 6-week-old NOD/SCID mice (10^6 ^cells/200 μl Matrigel/animal). Tumors were produced and as shown in Figure 5, all the tumors were GLUT1 positive indicating an IH cell of origin. Tumors exhibiting a proliferative cell phase were positive for CD31, an endothelial cell marker. These results demonstrated that IH stem cells can form hemangiomas *in vivo*. Tumors visible on day 10 exhibited mostly proliferative endothelial cells after H & E staining. Twenty days after injection, both blood vessels and fatty tissue became visible in H & E sections although proliferating cells were still present. By day 30, most of the cells injected into the animal had differentiated, and tumor involution occurred, with a high percentage of fatty tissue present (See Figure 5). This 30 day cycle of *in vivo *tumorigenis in mice represents the 5-9 year process seen in human cases of IH. The apparent faithful replication of hemangioma growth in this IH stem cell-induced *in vivo *hemangioma mouse model suggests that this system can be used as a tool to discover novel therapeutics specifically targeting IHs.

## Discussion

Infantile hemangiomas are the most common tumors of childhood and the head and neck region is most commonly affected. They may grow rapidly and cause destruction of normal anatomic structures, leading to severe disfigurement. Some of these tumors respond to medical therapy with beta-blockers. However, when this therapy fails, surgical excision may be necessary. Noninvasive therapies to debulk or eliminate these tumors would be an important advance. The development of an *in vitro *cell culture system and an animal model should permit new insights into the biological processes involved in the development and pathogenesis of IH.

The natural history of rapid proliferation during early infancy and subsequent involution strongly suggests that normal growth factors are regulating the growth and expansion of these tumors. In contrast to many other tumors which do not regress, IH is not naturally capable of sustained tissue renewal or growth. There has been speculation that the placenta may be the source of cells that form the IH because of the similarities between the expression of specific proteins such as GLUT1, and RNA array expression profiles from IH and the placenta. While the present studies cannot support or refute the placental origin of IH stem cells, they show that stem cell markers such as SALL4 and CD133 can be identified in IH. The tumors from younger patients with more proliferative IH contain more cells immunochemically staining for these early stem cell markers than tumors excised from older children which have more adipose and fibrous connective tissue characteristic of the involuting IH. Based on this study, the involution process is accompanied by, and related to a decrease in the number of IH stem cells.

IH stem cells have been isolated from IHs surgically removed from patients and grown to purity as tumor spheres in selective media. When grown in stem cell media the fibroblasts that initially grow out of the minced tissue are depleted and the resulting cell population consists only of IH tumor stem cells, which form tumor spheres. Complex protocols for the purification of tumor stem cells have been described utilizing methods such as FACs sorting and immunomagnetic purification strategies [12]. In contrast, the preparation of purified stem cell from IH tumors described here by growth in selective media is quite simple.

The cells from the IH tumor spheres have been shown to express stem cell markers and GLUT1-positive staining (Figure 2). The tumor spheres can be dissociated into single cells and can undergo many generations of growth *in vitro*. The dissociated cells can be reseeded to form new tumor spheres. This extensive multi-generational growth after multiple rounds of subculturing supports the concept that the IH stem cells are not preprogrammed to age and die within the *in vitro *culture system.

IH tumor sphere cells also express VEGF. VEGF is commonly expressed by tumor cells, presumably due to hypoxia, and aids in creating an optimal environment for growing tumors by increasing angiogenesis. Anti-VEGF therapies, including monoclonal antibodies such as bevacizumab (Avastin), antibody derivatives such as ranibizumab (Lucentis), or orally-available small molecules that inhibit the tyrosine kinases stimulated by VEGF: sunitinib (Sutent), sorafenib (Nexavar), axitinib, and pazopanib, have become well accepted chemotherapeutic agents. The sensitivity of IH tumor sphere cells to rapamycin implies that anti-VEGF therapy should be tested in the animal model described here, with potential eventual application for therapy.

When the tumor spheres are replated in medium containing serum, the spheres show an outgrowth of cells that are endothelial cells and their precursors (Figure 2), suggestive of the angiogenic differentiation seen in IH tumors. Cells derived from tumor spheres have been placed subcutaneously in immune-suppressed mice where they form tumors that recapitulate the histological pattern of IH removed from patients. The tumors removed from the inoculated mice stained with GLUT1 and CD31 confirming that they are of human origin and are similar to human IHs (Figure 5).

These IH xenografts were removed from the mice and studied at 10, 20 and 30 days post inoculation. Histological studies showed that at 10 days there was a tumor formed that was composed of proliferating endothelial cells and blood vessels consistent with a proliferative IH, at 20 days there was less proliferation and some fat and fibrous connective tissue consistent with the beginning of involution, at 30 days most of the vascular tissue was gone and there was a predominance of adipose and connective tissue (Figure 5). This temporal sequence mimics the natural course IH undergoes in humans.

The development of a mouse xenograft of IH and the establishment of an *in vitro *tissue culture system for propagating IH stem cell will be critical for assessing both host effects and the efficacy of drug therapies. The stem cell of the IH can now be studied for genetic aberrations as well as epigenetic modifications caused by the loss or gain of host growth factors or exogenous agents.

## Competing interests

The authors declare that they have no competing interests.

## Authors' contributions

All authors are accountable for the integrity of the research results. TMO, MW and YM are responsible for the conception of the research and DX, TMO, AS, TCF and JY are responsible for the execution and for data collection; JY, LMF, DCW, MM, MW and YM wrote the paper with contributions from the other authors. All authors read and approved the final manuscript.
